# Olympic fencing medallists are increasingly clustering around an age that has hardly changed (1896–2024)

**DOI:** 10.3389/fspor.2026.1938745

**Published:** 2026-10-07

**Authors:** Novrizal Achmad Novan, Edina Kardos, Zsolt Nemcsik, Leonidas Petridis

**Affiliations:** 1 School of Doctoral Studies, Hungarian University of Sports Science, Budapest, Hungary; 2 Faculty of Sport and Health Education, Universitas Pendidikan Indonesia, Bandung, Indonesia; 3 Department of Kinesiology, Hungarian University of Sports Science, Budapest, Hungary; 4 Department of Combat Sports, Hungarian University of Sports Science, Budapest, Hungary; 5 Research Centre for Sport Physiology, Hungarian University of Sports Science, Budapest, Hungary

**Keywords:** age at medal winning, age variability, career trajectory, combat sports, historical trends, sex differences

## Abstract

Fencing has been contested at every modern Olympic Games since 1896, giving it one of the longest continuous records in sports and an opportunity to examine whether an optimal age at medal-winning exists and whether it has changed. We examined every individual Olympic fencing medallist from 1896 to 2024, resulting in 379 medals won by 286 athletes across foil, épée, and sabre. Of these, the available birth date records of 282 athletes (>95% of the total) were retained, resulting in 375 medals with a date of birth resolved to the day of each event, and the analyses based on those. Across the full 128 years, the average medallist was 28.8 years old, and gold, silver, and bronze medallists were the same age as one another (*p* = 0.195). The mean age of male medallists changed little, falling by approximately one-third of a year per decade (*p* = 0.006), with no discontinuity at the end of the amateur era in 1972. However, the change pertained to the range of ages rather than the mean. Early medallists ranged from 16.8 to 50.0 years, and no male athlete has won an Olympic fencing medal beyond the age of 42 in the past 50 years. Ages were approximately 1.5 times as variable before 1972 as after it, and the narrowing occurred in foil, épée, and sabre alike. In the past two decades, the age of medal winners was concentrated in a narrow band: the mean age of medal winners was 27.7 years, and 50% all medals were won between 24.1 and 30.8 years. That band was similar across foil, épée, and sabre, and similar for women and men. Among Olympic medallists, therefore, the age at which the medals are won has not shifted; what has diminished is the winning of medals far from it. However, whether the same holds below the podium is not addressed by these data.

## 1 Introduction

Since the first modern Olympics in Athens in 1896, fencing has been contested at every edition of the Games, one of only five sports with such an uninterrupted presence (International Fencing Federation, https://fie.org/fie/history). It has developed over this period into three weapon disciplines, foil, épée, and sabre, with distinct sets of rules and tactical demands (1). This technical–tactical characteristics situate fencing between two contrasting age-related trajectories. The explosive neuromuscular capacities relevant to elite combat performance, including movement speed and reactive strength, peak in early adulthood and decline progressively over the decades that follow (2). In contrast, the perceptual-cognitive resources central to fencing, including anticipation, pattern recognition, and tactical decision-making, separate experienced experts from less skilled competitors and favour athletes who have accumulated years of competition (3, 4). These two trajectories generate competing expectations regarding when elite performance should occur. Comparative Olympic data support this intermediate position, as fencing medallists are not among the youngest combat-sport competitors, and their mean age at medal winning is higher than that in striking disciplines such as boxing and taekwondo (5).

However, it remains unclear which of these demands determines the age of elite success in fencing. Allen and Hopkins (6) reviewed the peak competitive performance age across Olympic sports and established consistent age-trajectory patterns for events dominated by physical capacities. The authors also reported that the available data were insufficient to characterise mixed and skill events, which contains technical–tactical disciplines such as fencing. The available evidence for mixed and skilled disciplines remains fragmented. Most studies examine a single Olympic Games or a restricted timeframe (7, 8) and typically report pooled averages without testing whether weapon discipline or sex produces a distinct age pattern within the sport (9). Whether age patterns in Olympic fencing behave the same way and whether they have remained constant has not been examined across the full span of the modern Olympic era.

Reading age patterns over the modern Olympic era requires attention to the conditions under which athletes prepared and competed because those conditions determine which of the two trajectories weighs more heavily. Olympic competition from 1896 onwards has occurred against substantial changes in the organisation of elite sports, including the documented transition from amateurism to professional structures across the mid- to late-twentieth century (10, 11). A standardised preparation system, with formalised training and selection, would be expected to shift the age of elite performance towards the physiological window rather than towards older ages associated with a longer accumulation of competitive experience. Analyses of Olympic medallist data spanning more than a century have reported shifts in the age at which athletes reach the podium across decades for several event categories (12). The consequences of these broader transitions remain unexamined for technical–tactical sports such as fencing.

No study of fencing has yet combined weapon, sex, and historical era in one dataset. The present study analysed the age at which medals were won at every Olympic Games from 1896 to 2024 and asked whether that age has changed across the modern era or has remained fixed. Three further questions followed: how the ages of medallists are distributed overall and whether gold, silver, and bronze differ; whether each of the three weapons carries its own age pattern; and whether sex and weapon shape that pattern jointly. One point deserves to be stated at the outset. A difference in age between two historical periods can arise from processes of quite different shapes, and comparing periods can reveal that a difference exists without revealing what produced it. The present study, therefore, sought to characterise the form of any change rather than assume it, distinguishing abrupt transitions at specific historical boundaries from changes that occur more gradually.

## 2 Methods

### 2.1 Study design

This retrospective cross-sectional study analysed all individual Olympic fencing medallists across the modern Olympic era, from the first Games in Athens in 1896 to the most recent Games in Paris in 2024, covering both sexes and the three weapon disciplines of foil, épée, and sabre. Because every individual medallist across this period was included, the dataset constitutes the complete target population rather than a sample drawn from it, a distinction that shapes the analytical approach throughout.

This study exclusively analysed publicly available, non-identifiable historical records of Olympic competition outcomes. As no direct participant contact, intervention, or collection of primary or identifiable data was involved, the study did not constitute human subjects research and did not require institutional ethics review.

### 2.2 Data sources and collection

For each athlete, the data collected from publicly available sources comprised their full name, year of birth, sex, weapon discipline, medal type (gold, silver, or bronze), and Olympic event. Two primary sources were used: olympics.com (https://www.olympics.com) and olympedia.org (https://www.olympedia.org). Records were cross-validated between the two sources, and discrepancies were referred to complementary public records such as the national Olympic committee archives and official tournament reports. The date of birth was retrieved at full calendar resolution from Olympedia for every medallist and cross-checked against the national Olympic committee and international federation records wherever the two sources disagreed. Full birth records (day, month, and year) are available for 375 of the 379 medal observations. The four remaining medallists, all from the 1896 to 1924 period, had records for birth year only and were excluded from every analysis of age.

Some athletes competed under different names throughout their careers, for example, following marriage or because of transliteration differences between alphabets. Athlete identity was, therefore, defined by the Olympedia athlete identifier rather than by name, which resolves marriage names, transliteration variants, and inconsistent diacritics to a single record. The date of birth, nationality, and weapon discipline were used to confirm each match. The 379 medal observations belong to 286 distinct athletes on this definition, of whom 282 had a precise date of birth to enter the analyses of age.

### 2.3 Inclusion and exclusion criteria

Inclusion required an individual medal (gold, silver, or bronze) in foil, épée, or sabre at any Games from 1896 to 2024, available biographical data (name, year of birth, and sex), and a verifiable medal record from official sources. Athletes were excluded if their year of birth could not be verified or if cross-source discrepancies could not be resolved.

The analysis was restricted to medallists rather than a broader finalist pool for two reasons. The first is definitional. First, second, and third places carry the same meaning at every Games in the record, whereas the size of the final round and the method by which places below the podium were settled did not remain constant across 128 years; therefore, a top-eight series would not provide a consistent basis for comparison across periods. The second is documentary. The present dataset was compiled for medal positions, and birth dates for competitors placed below the podium were not recorded with the same completeness across the historical span. Extending the population downwards would therefore admit missing data whose extent varies with era, and the era is precisely the dimension under study. Restricting the analysis to medallists holds the inclusion rule fixed across the entire period, at the cost of confining every conclusion to the medal-winning population. Every statement in this article is about Olympic fencing medallists and should not be read as describing elite fencers in general. The Olympic Games were chosen over the World Fencing Championships because only the Olympic series spanned the entire period under study. Fencing has been featured at every Olympic Games since 1896.

The World Championship Series began in 1921 with one event, men's épée. Men's sabre followed in 1922, men's foil in 1926, women's foil in 1929, women's épée in 1988, and women's sabre in 1999 (13). No championships were held between 1939 and 1946, and for much of the twentieth century, the events on the Olympic programme were omitted from the championships staged in Olympic years. A championship series assembled from these records would begin 25 years into the period, would contain different weapons in different decades, and would be interrupted for 8 years in the middle. The Olympic record carries none of these features: the same three weapons at the same 4-year interval from the first modern Games to the most recent. Team events were excluded to avoid double-counting of athletes who also won individual medals. For athletes who medalled across multiple editions, each medal-winning event was treated as a separate observation, yielding 379 observations from 286 unique athletes, because each medal represents a discrete competitive achievement at a distinct career stage rather than a repeated measurement of an underlying value. This decision creates statistical dependence among observations from the same athlete, which is addressed directly in the analysis rather than assumed away. Its robustness was further evaluated through a sensitivity analysis at the athlete-Olympic aggregated level (*N* = 365), which collapsed the 10 instances of an athlete winning more than one individual medal at the same Games.

### 2.4 Variables and data processing

The dependent variable was age at medal winning, calculated as decimal age, following the convention used in previous research on Olympic age of performance (5) and its application to fencing (8). Decimal age is the interval in days between an athlete's date of birth and the reference date of the event at which the medal was won, divided by 365.25. The reference date was the closing date of the individual event, obtained from Olympedia for all 126 individual events across the 30 Games. Earlier work has used the opening date of the Games (5), which is adequate for the modern programme but not for the early one, because the Games of 1900, 1904, 1908, and 1920 each extended over more than 4 months, whereas their fencing programmes did not. The largest discrepancy between the two conventions in the present record was 125 days. Two alternative decimal conventions, division by the mean Gregorian year and completed years plus the elapsed fraction of the current anniversary year, were computed in parallel; the largest disagreement between any two of the three is 1.45 days, so the choice of convention does not affect any result reported here. The independent variables were medal type, weapon discipline, athlete sex, and Olympic year.

To examine how age patterns changed across the modern Olympic era, the Olympic year was also operationalised as a five-category era variable. Defining temporal categories in long-period Olympic datasets follows the established precedent in age-performance studies (12, 14, 15). The era boundaries used here reflect two documented sources of discontinuities. The first is the cancellation of the Olympic Games during the World Wars, which led to the suspension of the 1916, 1940, and 1944 editions of the Games. The second is the transition from amateurism to professional structures in the mid- to late-twentieth century (10, 11). On these grounds, five eras were defined: Early Olympic (1896–1912), interwar (1920–1936), post-war (1948–1968), modern training (1972–2000), and contemporary (2004–2024).

### 2.5 Statistical analysis

All analyses were conducted using Python version 3.12 and the pandas, scipy, statsmodels, and pingouin libraries. No decision threshold was applied to the *p*-values, and the language of statistical significance was not used. The dataset enumerates the target population rather than a sample drawn from it; therefore, no inference is made from a sample to a wider set of medallists. The record is nonetheless one realisation of a process that could have turned out differently: injuries, boycotts, and single touches decide podium places. A single touch is one scoring hit; an Olympic fencing bout can be decided by a margin of one, so a single exchange can separate a medallist from a fencer who leaves without a medal. Confidence intervals, bootstrap intervals, and *p*-values are reported as statements about that process, not about sampling from a population of athletes. Descriptive statistics (mean, standard deviation, 95% confidence interval, and range) summarised the age at medal winning overall and by medal type, weapon, sex, and era. Because the normality assumption of analysis of variance concerns the model errors rather than the raw scores, it was assessed on the model residuals using the Shapiro–Wilk test with *Q*–*Q* plots; homogeneity of variance was assessed using Levene's test. The *post-hoc* and robust procedures reported in the Results section were selected conditionally on these checks rather than fixed in advance, so that each comparison used a method matched to whether the variances were equal. Effect sizes were reported as eta squared (*η*^2^) for one-way analyses and partial eta squared (*η*^2^*_p_*) for two-way analyses, with 95% confidence intervals for all estimates.

Age differences across medal types and weapon disciplines were examined using one-way analysis of variance. Tukey HSD was applied where Levene's test indicated homogeneous variances, and Games–Howell was used where it did not. The joint influence of sex and weapon discipline was examined using a two-way analysis of variance with Type III sums of squares, chosen because the design was unbalanced and Type III estimates each effect, adjusting for the others; interactions large enough to warrant decomposition were examined using estimated marginal means with Bonferroni adjustment.

Temporal change among male medallists was the primary analysis and was conducted as a sequence of tests. The first tested whether the mean age differed across the five eras (one-way analysis of variance). Because the spread of ages differed markedly between eras, which made the standard *F* test unreliable, the omnibus comparison used a test tolerant of unequal variance (Brown–Forsythe), followed by Games–Howell for the pairwise differences. Brown–Forsythe was preferred to the Welch alternative because it is more robust when group sizes differ across eras. It was implemented directly from its defining formula because it was not available in the libraries used.

An omnibus difference across eras establishes that the mean age was not constant, but leaves the form of that difference open. The Olympic year was, therefore, treated as a continuous predictor, and a set of nested piecewise-linear regression models was compared. Each model contained a linear year term and, where specified, one or more hinge terms of the form max(0, year – knot). The hinge term allows the slope to change at the candidate breakpoint. The function remains continuous across this point. Candidate knots were placed at 1914, 1939, 1972, and 2000, the same documentary boundaries used to define the era variable. The resulting models were compared with an intercept-only model and with a single-slope linear model. Ranking was performed using the Akaike information criterion. Because several athletes contributed more than one medal, standard errors were clustered on athlete identity in the piecewise-linear models, dispersion regression, and female sabre trend. This comparison tests whether the change in the mean age manifested as a discontinuity at any documented boundary or as a continuous trend across the entire period. Age was also compared between the pre-1972 and post-1972 periods using Welch’s *t*-test and Cohen's *d*. This contrast is reported as a description of levels, not as a test of form.

A change in the mean may be accompanied by a change in spread; therefore, dispersion was analysed as a separate outcome. The ratio of the two standard deviations was reported with a percentile bootstrap 95% confidence interval based on 10,000 resamples, in which athletes, rather than individual medals, were the unit resampled. To establish whether any contraction in spread was gradual or abrupt, the absolute deviation of each medallist's age from the overall mean was regressed on the Olympic year, again with clustered standard errors.

Because several athletes contributed more than one medal, the assumption of independence underlying the analysis of variance and regression procedure is violated. An unconditional linear mixed model with athlete-level random intercepts, fitted by restricted maximum likelihood, quantified the share of variance in age at medal winning that lay between athletes rather than within them. Whether the random intercept was warranted was tested by comparing the model against an ordinary least-squares model with the same fixed effects. Because the null value of a variance component lies on the boundary of the parameter space, the null distribution of the likelihood-ratio statistic is not a chi-square distribution on the number of parameters removed. It is a 50:50 mixture of a point mass at zero and a chi-square distribution with one degree of freedom, and the *p*-value was computed on that basis. The distribution of random intercepts deviates from normality because most medallists appear only once. This does not bias the fixed-effect estimates, and every principal inference is reported alongside a procedure that does not rely on that assumption.

Medals won by the same athlete are not independent observations; therefore, the intraclass correlation and the design effect were calculated (16). The clustering proved shallow, with most medallists winning a single medal, and the resulting design effects were of a size conventionally treated as moderate rather than severe.

The mixed model is reported as a primary analysis rather than a robustness check, and standard errors clustered on athlete identity are applied to the piecewise-linear models, the dispersion regression, and the female sabre trend. Analysis of variance, Levene's test, and Brown–Forsythe cannot accommodate clustered errors and are reported without them. For the two comparisons on which the substantive conclusions rest, the era effect among men and the sex-by-weapon interaction, an athlete-level counterpart is reported beside the medal-level results.

To establish whether the era effect was system-wide or weapon-specific, a two-way analysis of variance crossed era with weapon discipline for male medallists; the era × weapon interaction provided a direct test of weapon specificity. Among female medallists, era effects were examined using a one-way analysis of variance across the four available eras. This was considered *a priori* as underpowered rather than a null result because women's participation began later and expanded through the staged introduction of weapons (foil in 1924, épée in 1996, and sabre in 2004). Consequently, the resulting temporal depth was shallow and confounded with weapon introduction. As a secondary, hypothesis-generating analysis, the age trajectory of female sabre medallists was modelled by linear regression of age on Olympic year using individual medallists rather than edition means, because edition means understated the scatter around the trend. Because a short series cannot separate ageing within athletes from a change in who reaches the podium, the trend was decomposed into within-athlete and between-athlete components, and the analysis was repeated, counting only each athlete's first medal.

No family-wise correction was applied across the separate test families, each of which addressed a distinct, pre-specified research question; within-family error was controlled for multiple comparisons. Because the dataset enumerates Olympic fencing medallists rather than sampling them, no *a priori* power analysis was performed, as there was no sampling error against which to protect. The confidence intervals reported for the dispersion ratio and regression slopes quantify a different uncertainty, namely, how far the observed pattern could have differed had the same development system produced a different set of podium places. Age computed from the year of birth alone carries an error of up to 11 months. The whole-year and decimal measures were therefore compared directly on the 375 analysed observations, and the direction of the bias introduced by rounding was derived analytically using Sheppard's correction (17, 18).

## 3 Results

### 3.1 Participant characteristics

Across 30 Olympic Games from 1896 to 2024, the dataset comprised 375 medal-winning observations from 282 unique athletes (male 69.6%; female 30.4%). The mean age at medal winning was 28.78 years (SD = 5.75; range: 16.8–50.0). The full descriptive breakdown by medal type, weapon discipline, sex, and era is presented in Table 1.

**Table 1:** Age at medal winning by medal type, weapon discipline, and historical era, stratified by sex.

Variable	Category	*n*	*M* ± SD	95% CI	Range
Medal type	Gold	126	28.09 ± 5.48	[27.13, 29.06]	16.79–41.86
	Silver	125	28.86 ± 5.72	[27.85, 29.87]	18.14–50.01
	Bronze	124	29.40 ± 6.03	[28.33, 30.48]	18.50–50.01
Weapon	Foil	157	27.94 ± 5.83	[27.02, 28.86]	17.62–50.01
	Épée	111	29.51 ± 5.63	[28.45, 30.57]	16.79–50.01
	Sabre	107	29.25 ± 5.67	[28.17, 30.34]	18.50–42.38
Era (male)	Early Olympic (1896–1912)	37	29.92 ± 8.23	[27.17, 32.66]	16.79–50.01
	Interwar (1920–1936)	44	31.17 ± 5.42	[29.52, 32.81]	21.33–41.86
	Post-war (1948–1968)	54	31.36 ± 6.10	[29.70, 33.03]	19.84–44.84
	Modern training (1972–2000)	72	27.42 ± 4.28	[26.41, 28.42]	19.01–36.50
	Contemporary (2004–2024)	54	27.62 ± 4.76	[26.32, 28.92]	20.62–41.63
Era (female)	Interwar (1920–1936)	12	27.01 ± 6.56	[22.84, 31.18]	17.62–41.20
	Post-war (1948–1968)	18	29.54 ± 6.99	[26.07, 33.02]	18.14–45.20
	Modern training (1972–2000)	30	26.54 ± 3.86	[25.10, 27.98]	19.18–34.01
	Contemporary (2004–2024)	54	27.85 ± 5.08	[26.46, 29.23]	18.50–38.77

*N* = 375 medal observations from 282 unique athletes. Four of the 379 medallists are recorded only to the year of birth and are excluded. Age is a decimal computed to the closing date of each event. CI, confidence interval. The female era stratum begins in the Interwar period, reflecting the staged Olympic introduction of women's fencing events.

Across the two most recent Olympic decades (2004 to 2024, *n* = 108 medal observations of both sexes), the age at medal winning averaged 27.73 years (SD = 4.90; median 27.00), with half of all medals won between 24.08 and 30.77 years and a full range of 18.50–41.63. The corresponding means were 27.48 in foil, 28.47 in épée, and 27.25 in sabre, and 27.62 for men and 27.85 for women.

### 3.2 Assumption checks

The residuals of both models deviated only slightly from normality (Shapiro–Wilk *W* = 0.97 for the sex-by-weapon model and *W* = 0.98 for the male era model), and the probability plots confirmed that these deviations were mild.

Spread was comparable across the sex-by-weapon cells [Levene's test *F*(5, 369) = 0.49, *p* = 0.783], so the standard estimates were retained for the analysis. The spread across historical eras differed materially, which required a more tolerant omnibus test for comparison.

### 3.3 Age differences by medal type and weapon discipline

Age did not differ by medal type [*F*(2, 372) = 1.64, *p* = 0.195, *η*^2^ = 0.009]. The difference across weapon disciplines was small [*F*(2, 372) = 2.95, *p* = 0.054, *η*^2^ = 0.016; Table 1].

### 3.4 Temporal change in mean and age dispersion

#### 3.4.1 Male medallists

Ages were spread far more widely in some eras than in others [Levene's test *F*(4, 256) = 6.59, *p* < 0.001], with the earliest era being the most varied (SD = 8.23 years) and the Modern Training era the least (SD = 4.28 years). The mean age differed across the five eras [Brown–Forsythe *F*(4, 154.69) = 5.62, *p* < 0.001; *η*^2^ = 0.090]. The difference lay between the older and the newer halves of the century rather than between adjacent eras: medallists of the Interwar and Post-War years were 3.5–3.9 years older than those of the Modern Training and Contemporary eras (Games–Howell, all *p* ≤ 0.007), while the three early eras did not differ among themselves, nor did the two recent ones (all *p* > 0.360).

Split at 1972, the two halves of the century differ markedly: medallists before that year were 3.4 years older than those after it [*M* = 30.9, SD = 6.53, *n* = 135 vs. *M* = 27.5, SD = 4.47, *n* = 126; Welch *t*(238.0) = 4.93, *p* < 0.001, *d* = 0.60, 95% CI (2.04, 4.76)]. No discontinuity was observed in 1972. Among men, age fell steadily across the whole century, at 0.36 years per decade [*p* = 0.006, 95% CI (−0.62, −0.10), *R*^2^ = 0.055], amounting to fewer than 5 years across the 128-year period. Allowing the trend to bend at 1972 did not describe the data better; it described them worse than a single unbroken line (ΔAIC = 9.25 against 7.63), and the bend itself was too small to distinguish from no bend (−0.23 years per decade, *p* = 0.619). Of the four documented boundaries tested, the one favoured by the data was not 1972 but 1914 (ΔAIC = 0.00, *R*^2^ = 0.089), and a model carrying all three of 1914, 1939, and 1972 improved on none of them (ΔAIC = 0.62).

The four documented boundaries are not the only candidates that the record admits. A search over every Olympic year as a possible breakpoint placed the best-supported change in slope at 1904, 68 years before the amateurism boundary; 1972 ranked 22 of the 26 candidates tested, and 14 of the 26 fitted worse than no breakpoint at all (Supplementary Table 2). The decline in medal-winning age was, therefore, gradual rather than a step change (Figure 1).

**Figure 1:**
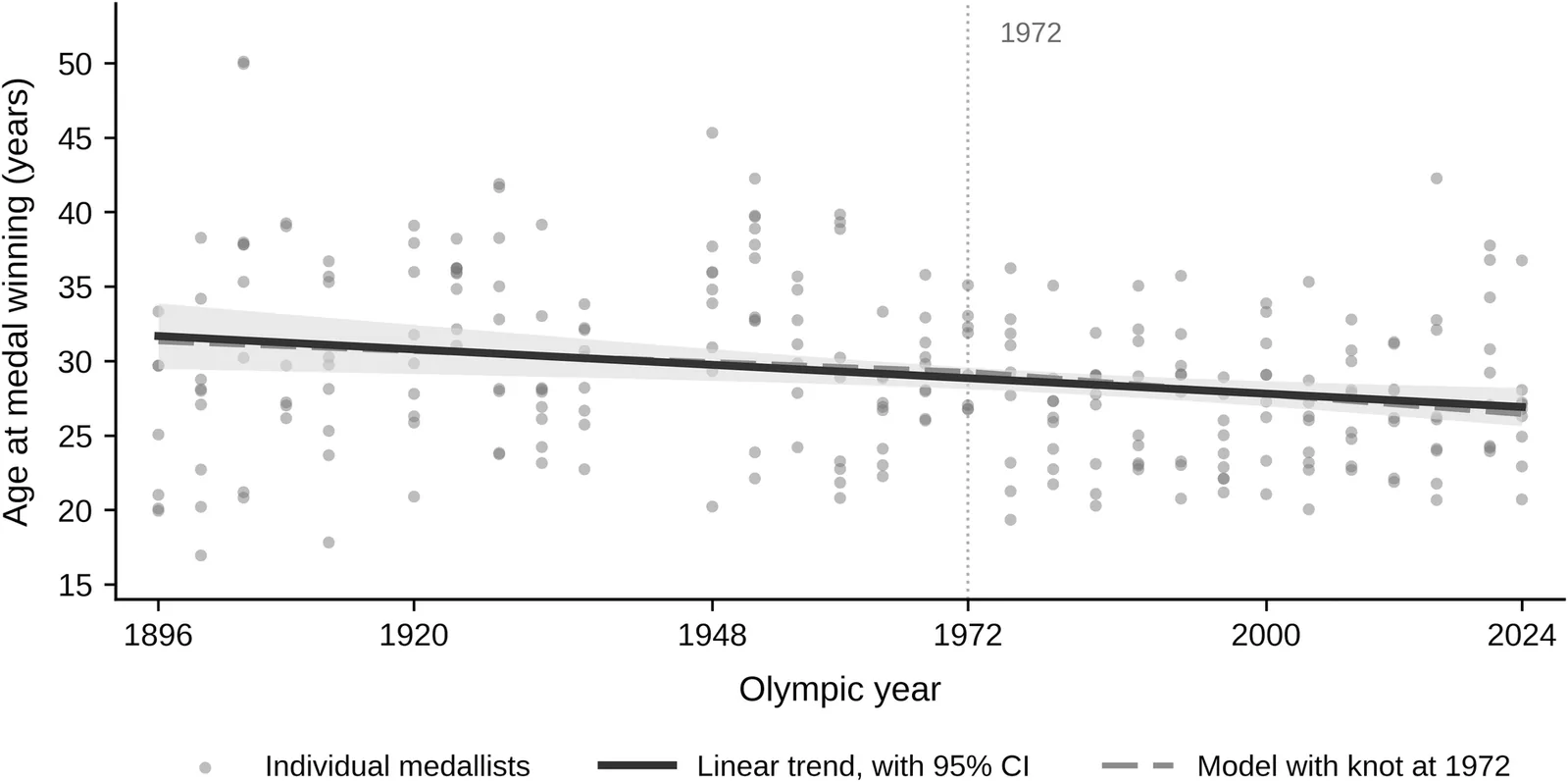
Age at medal winning among male Olympic fencers by Olympic year, 1896–2024.

The change was in the dispersion of ages rather than in the mean. Before 1972, the age of male medal winners was more variable than after [SD = 6.53 vs. 4.47 years; ratio 1.461, 95% CI (1.212, 1.765); Levene's test *W* = 18.22, *p* < 0.001]. The contraction was gradual, with the mean absolute deviation of age decreasing by 0.20 years per decade (*p* = 0.008). Across eras, the standard deviation fell from 8.23 years in the Early Olympic era to 4.76 years in the Contemporary era, though not monotonically, as the post-war generation (6.10) was more variable than the Interwar generation (5.42). The upper tail contracted the most: the oldest male medallist was 50.0 years in the early period and 36.5 years in the Modern Training era (Figure 2).

**Figure 2:**
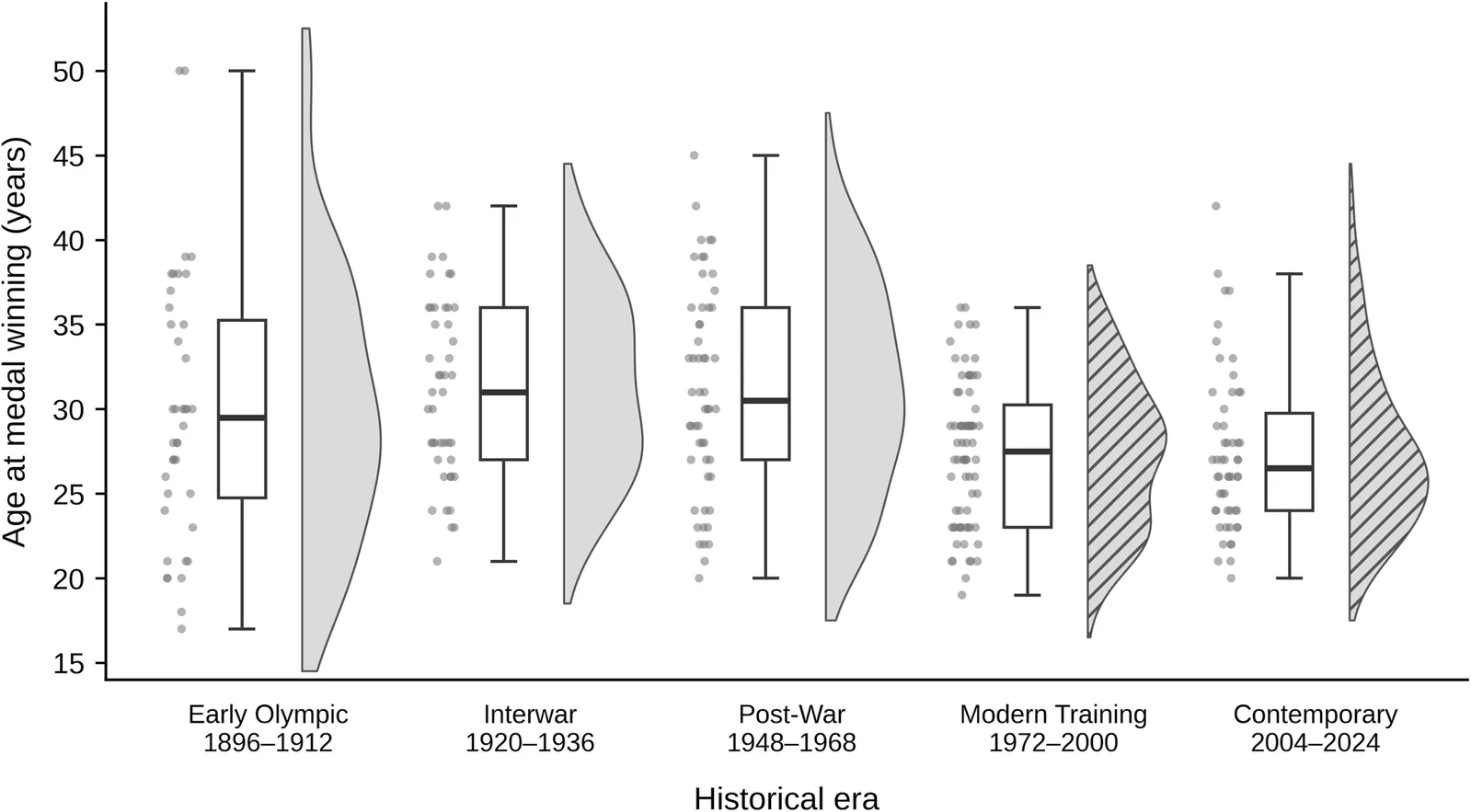
Distribution of age at medal winning across five historical eras for male medallists.

Among the 375 medallists, the mean discrepancy between whole-year and decimal age was 3.14 months, and the two measures correlated at *r* = 0.999. Rounding to whole years introduces an approximately uniform error to each observation, inflating the variance of each period by the same amount; adding a constant to two variances moves their ratio towards unity. Whole-year rounding, therefore, biases the dispersion ratio towards no difference and cannot manufacture a contraction: the ratio stands at 1.461 at decimal resolution against 1.440 at whole-year resolution for the same observations.

#### 3.4.2 Female medallists

Among women, the mean age did not change across the four eras in which they competed [−0.07 years per decade, *p* = 0.746, 95% CI (−0.50, 0.36)]. Of the models compared, the best supported was the model holding age constant; allowing a bend at 1972 did not improve the model (ΔAIC = 2.52 against the flat model, with the bend itself *p* = 0.279).

No era difference emerged among female medallists [*F*(3, 110) = 1.28, *p* = 0.285, *η*^2^ = 0.034].

The age of female medal winners moved in the same direction, although it was estimated too imprecisely to establish that it moved at all. The standard deviation fell from 6.83 years before 1972 to 4.75 years after (ratio 1.45, compared with 1.461 in men), and the oldest female medallist fell from 45.2 to 38.8 years. With only 30 female medallists before 1972 against 139 male medallists, the female confidence interval for the ratio ranges from 0.969 to 1.846 and includes the point of no change. Levene's test indicates a difference (*W* = 4.25, *p* = 0.042), but it does not account for the dependence among medals won by the same fencer; both results are reported rather than preferring one over the other. The female contraction is best read as consistent with the male contraction rather than being independently established.

### 3.5 Weapon-specificity of the era effect

Crossing era with weapon discipline, the main effect of era was retained [*F*(4, 246) = 6.33, *p* < 0.001, *η*^2^_p_ = 0.093], and the era × weapon interaction was negligible [*F*(8, 246) = 0.94, *p* = 0.486, *η*^2^_p_ = 0.030]. The narrowing, therefore, occurred in foil, épée, and sabre alike rather than in any single weapon.

### 3.6 Age differences by sex and weapon

#### 3.6.1 Sex × weapon interaction

A two-way analysis of variance with Type III sums of squares and effect coding returned a sex × weapon interaction [*F*(2, 369) = 3.47, *p* = 0.032, *η*^2^_p_ = 0.018]. Because the two factors interact, neither main effect describes the data independently. The sex term [*F*(1, 369) = 7.19, *p* = 0.008, *η*^2^_p_ = 0.019] averaged a negligible difference in foil and a substantial difference in sabre, and the weapon term [*F*(2, 369) = 1.33, *p* = 0.267, *η*^2^_p_ = 0.007] averaged across sexes whose orderings by weapon were not the same. Decomposition by estimated marginal means located the difference in sabre: female sabre medallists were 4.5 years younger than male medallists (25.53 vs. 30.01 years; Bonferroni-adjusted *p* = 0.007, *d* = 0.79). The sex difference was 0.05 years in foil and 1.4 years in épée (Figure 3). The robustness of the interaction was confirmed at the athlete-aggregated level [*N* = 365: *F*(2, 359) = 4.73, *p* = 0.009]. Female Sabre: A Countervailing Trend

**Figure 3:**
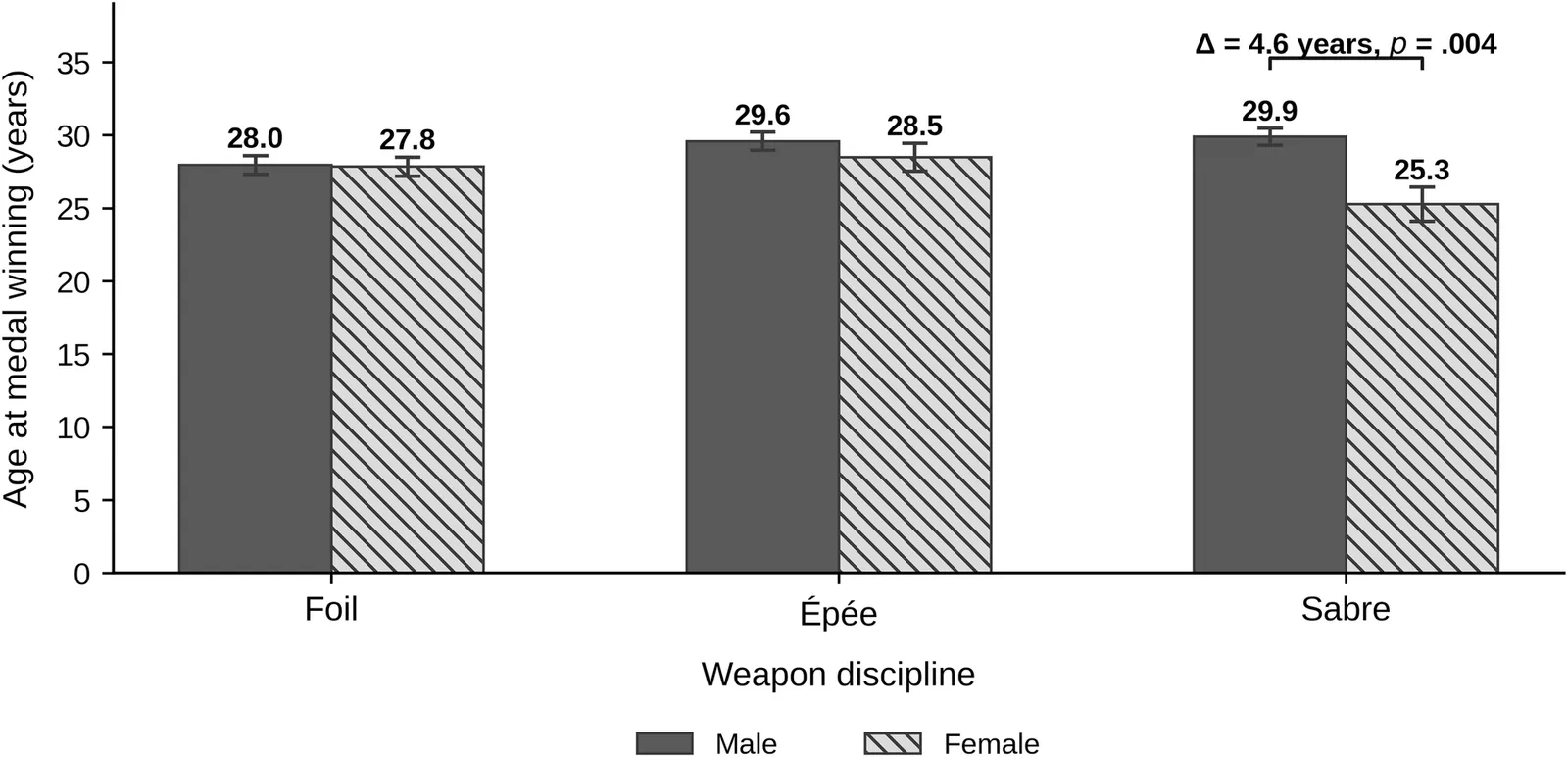
Age at medal winning by weapon and sex.

#### 3.6.2 Female sabre: a countervailing trend

The female sabre, the most recently introduced event and the youngest subgroup in the dataset, showed the opposite trend. The mean age of medallists increased across editions, from 22.49 years in 2004–2012 to 28.57 years in 2016–2024 (slope = 0.49 years per year, *R*^2^ = 0.54, *p* < 0.001, *n* = 18). The difference from male sabre medallists narrowed from 4.48 years across the entire period to 2.63 years in the most recent three editions.

The composition of the podium makes the difficulty concrete: the 18 medals were won by 11 athletes, and five of those athletes accounted for 12 of the 18 (Figure 4). The within-athlete slope was 0.998 years per year [95% CI (0.44, 1.56), *p* = 0.002] and the between-athlete slope was 0.401 years per year [95% CI (0.16, 0.64), *p* = 0.003]. When only each athlete's first medal is counted, the increase across the two periods is 3.46 years rather than 6.08 years. Approximately half of the increase is carried by the within-athlete component and half by the between-athlete component.

**Figure 4:**
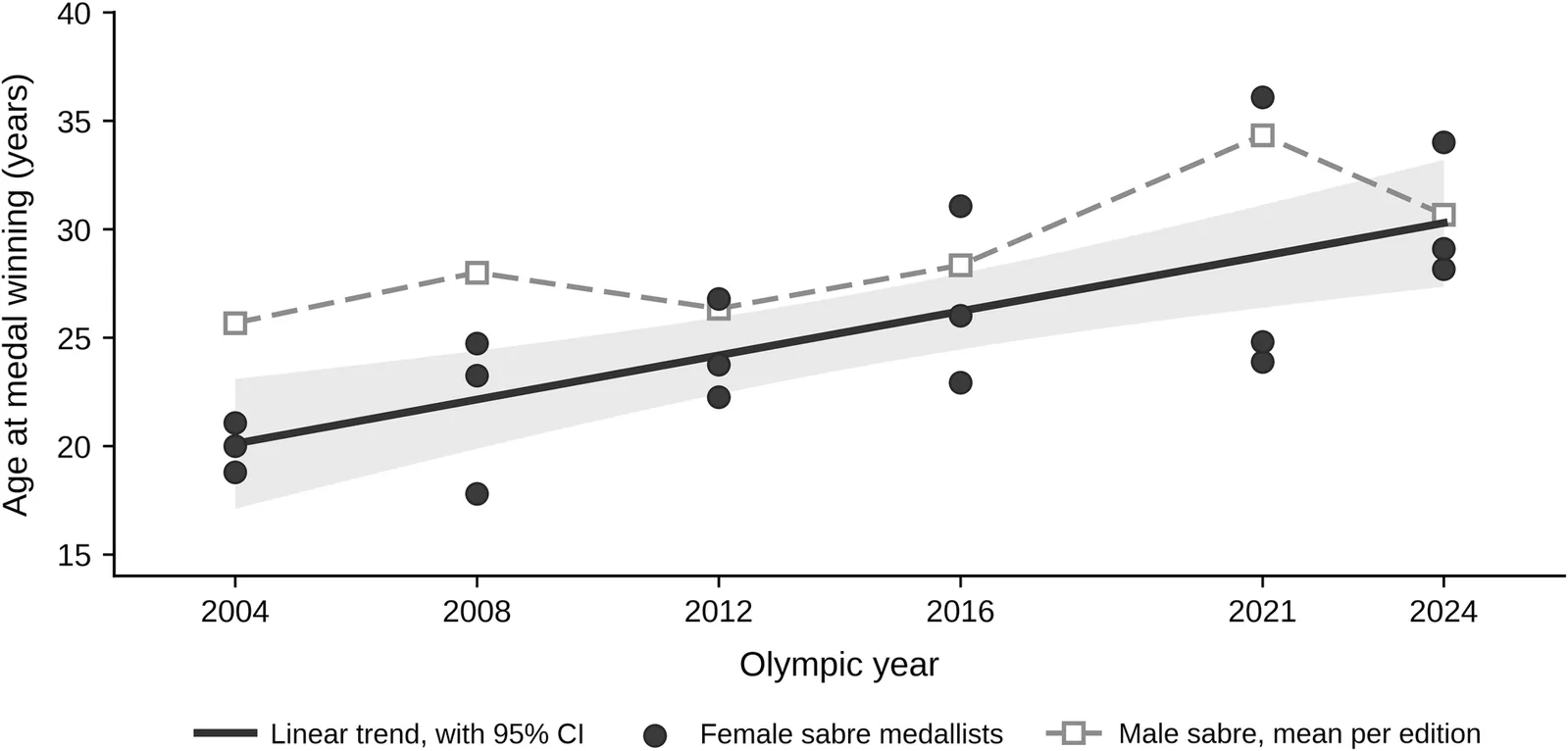
Age at medal winning among female sabre medallists by Olympic year, 2004–2024.

### 3.7 The team-only medallists

Restricting the analysis to individual events removes from the record every athlete whose Olympic fencing medals were won only in team competitions. This group was not small. Across the same 30 Games, 769 fencers won a team medal without ever winning an individual one. Because a team medal is won by a squad, the 308 team medals in the record generate 1,037 medal-winning appearances by these athletes. Together with the 286 individual medallists, they make up the 1,055 people who have won Olympic fencing medals, of whom the analysed population is 27.1%. The exclusion does not, however, remove team fencers from the analysis: 199 of the 286 individual medallists also won at least one team medal, so seven in 10 of the athletes analysed here are team medallists. The inclusion rule removes their team appearances, together with fencers who never won individually.

Team-only medallists were 0.53 years older on average than individual medallists [29.31 vs. 28.78 years; Welch *t*(668.1) = 1.51, *p* = 0.131, *d* = 0.09], and their ages were no more dispersed (SD 5.81 vs. 5.75; ratio 1.009; Levene *W* = 0.03, *p* = 0.869). Era by era, the two groups take a similar shape without being identical: the mean age peaks in the Interwar era among team-only medallists and one era later among individual medallists, both decline into the Modern Training era, and both increase slightly in the most recent one, while dispersion narrows in both towards the modern period (Supplementary Table 1). They are not similar throughout. In the Early Olympic era, the team-only medallists were the more uniform group of the two (SD = 5.85 against 8.23), and it is that difference which determines what happens when they are restored. Including the excluded medallists in the male record maintains the central finding standing while slightly softening it: the ratio of standard deviations across the 1972 boundary is 1.450 with them included [95% CI (1.343, 1.574)] compared to 1.461 without them The interval narrows because the number of observations quadruples.

### 3.8 Non-independence of repeated medals

Because several athletes won more than one medal, the dependence among observations was quantified rather than assumed (Table 2). Its structure is shallow. Among male medallists, on whom the temporal analyses rest, 156 of 200 won a single medal, 30 won twice, 11 won three times, and three won four times; no man won more than four. Across the whole record, 216 of the 282 medallists with a recorded date of birth won once, and the maximum was five wins. Within that structure, 48.8% of the variance in age at medal winning lay between male athletes rather than within them, and 38.6% lay across the whole record. The random intercept was warranted on both definitions (boundary likelihood-ratio test, male LR = 25.92, df = 1, *p* < 0.001; whole record LR = 23.18, df = 1, *p* < 0.001).

**Table 2:** Inferential statistics for age at medal winning across all tests.

Effect/test	Test statistic	*p*	Effect size
Medal type (one-way ANOVA)	*F*(2, 372) = 1.64	0.195	*η*^2^ = .009
Weapon discipline (one-way ANOVA)	*F*(2, 372) = 2.95	0.054	*η*^2^ = .016
Sex × weapon (two-way, Type III)	*F*(2, 369) = 3.47	0.032	*η*^2^_p_ = .018
Sex (two-way main effect, Type III)	*F*(1, 369) = 7.19	0.008	*η*^2^_p_ = .019
Weapon (two-way main effect, Type III)	*F*(2, 369) = 1.33	0.267	*η*^2^_p_ = .007
Male era (one-way ANOVA)	*F*(4, 256) = 6.30	<0.001	*η*^2^ = .090
Male era (Brown–Forsythe)	*F*(4, 154.69) = 5.62	<0.001	—
Male era (two-way main effect)	*F*(4, 246) = 6.33	<0.001	*η*^2^_p_ = .093
Era × weapon (male, interaction)	*F*(8, 246) = 0.94	0.486	*η*^2^_p_ = .030
Female era (one-way ANOVA)	*F*(3, 110) = 1.28	0.285	*η*^2^ = .034
Level difference pre/post-1972 (Welch t)	*t*(238.0) = 4.93	<0.001	*d* = 0.60
Dispersion pre/post-1972 (Levene)	*W* = 18.22	<0.001	SD ratio = 1.461
Within-athlete clustering, male (ICC)	—	—	ICC = .488
Within-athlete clustering, whole record (ICC)	—	—	ICC = .386
Random intercept warranted, male (boundary LRT)	LR = 25.92, df = 1	<0.001	DEFF = 1.303
Random intercept warranted, whole record (boundary LRT)	LR = 23.18, df = 1	<0.001	DEFF = 1.261
Sensitivity, male era at the athlete-by-games level	*F*(4, 246) = 5.90	<0.001	*N* = 251
Individual vs. team-only medallists (Welch *t*)	*t*(668.1) = 1.51	<0.131	*d* *=* 0.09
Individual vs. team-only dispersion (Levene)	*W* = 0.03	0.869	SD ratio = 1.009

*N* = 375 unless otherwise stated. Age is a decimal. The Brown–Forsythe test is reported for the male era comparison because Levene's test indicated a heterogeneity of variance across eras [*F*(4, 256) = 6.59, *p* < 0.001]. Type III sums of squares are used for factorial analyses with effect coding. The ICC is derived from an unconditional linear mixed model with athlete-level random intercepts; the boundary likelihood-ratio test compares that model against ordinary least squares with the same fixed part, referred to as a 50:50 mixture of a point mass at zero and a chi-square distribution on one degree of freedom. DEFF is the design effect, 1 + (*m¯w* − 1) × ICC, where m¯w is the size-weighted mean number of medals per athlete, used because athletes contribute unequal numbers of medals. Team-only medallists are reported for comparison only and are not part of the analysed population; the analysed comparison rests on 1,034 appearances by 766 athletes of the 1,037×769, three of whom lack a date of birth at full resolution. *η*^2^, eta squared; *η*^2^_p_, partial eta squared; *d*, Cohen's *d*. *p*-Values <0.001 are reported as *p* < 0.001.

The consequence of precision can be stated exactly. Because athletes contribute unequal numbers of medals, the design effect is computed from a size-weighted mean rather than a simple one, giving 1.303 among men and 1.261 across all records. The 261 male observations, therefore, carry the information of approximately 200 independent observations, and the 375 observations of approximately 297: a loss of approximately one-quarter and one-fifth, respectively. Inflation of this size is moderate rather than negligible. Two procedures that do not assume normally distributed random intercepts are reported alongside: the Brown–Forsythe test and an analysis giving each athlete one age per Games [*N* = 251 for men, *F*(4, 246) = 5.90, *p* < 0.001].

## 4 Discussion

Across the full 128-year record, medallists won at a mean age of 28.78 years (SD = 5.75, range: 16.79–50.01). A sport that rewards both youthful speed (2) and accumulated tactical judgement (3, 4) should produce medallists of widely different ages, and the pooled distribution is consistent with this expectation. This pooled value is an artefact of averaging. It does not match any single period because it combines a broad early distribution with a narrow modern one. When divided into periods in which the medals were won, the record shows two changes: the mean age declined gradually, and the dispersion around it narrowed. The mean is discussed first, followed by the dispersion.

Among male medallists, the mean age declined gradually across the period, by approximately 0.36 years per decade, with no discontinuity at the end of the amateur era. A segmented model that allowed the trend to change slope in 1972 fitted the data less well than a single linear trend. The estimated change in slope was too small to be distinguished. The 3.4-year difference between the pre- and post-1972 halves of the record is, therefore, not evidence of an abrupt transition. A steady linear decline, divided at any point and averaged on either side, produces a difference of this size as an arithmetic consequence. Of the four documented boundaries, the data favoured 1914, and a search over every Olympic year placed the best-supported change earlier, at 1904. Both fall in the sparsest part of the record, and the 1904 solution rests on the 13 male observations that precede it.

The more substantial change concerned the dispersion of medal-winning age rather than its mean value. Ages were approximately 1.5 times as variable before 1972 as after it [SD: 6.53 vs. 4.47 years; ratio of standard deviations 1.461, 95% CI (1.212, 1.765)]. The oldest age at which a medal was won fell from 50.0 to 36.5 years in the Modern Training era, and it stood at 41.6 in the contemporary era. As with the mean, this contraction was gradual, with dispersion narrowing by approximately 0.20 years per decade and no abrupt reduction at any single point. The trend was not steady throughout because the post-war cohort was more variable in age than the preceding interwar cohort.

These findings indicate concentration rather than a shift. The age at which medals are won has changed little; however, what has diminished is the winning of medals outside the central age range. A systematic pathway into elite sports need not change the age at which medals are most often won. It would remove the route by which a gifted amateur might reach the podium at 45 or 50 under a loosely organised system. The older medallists in the early record are consistent with a system that could have admitted them. Their disappearance coincided with the dismantling of amateurism and the rise of formal, state- and federation-supported development structures through the middle and later 20th century (10, 11, 19).

Against the male trajectory, the female sabre is the only series in the data that moved significantly. The event was included in the Olympic programme in 2004. Across six editions, the mean age of its medallists increased by approximately 6 years, and the difference from male sabre medallists narrowed from 4.5 to 2.6 years.

An earlier version of this work read that the rise is evidence of a consolidating preparation system. A second account requires fewer assumptions. The second account requires fewer assumptions. A small group of competitors has dominated the event since its introduction, and a competitor who medals at successive games is necessarily older at each. The decomposition reported in the results earlier splits the rise approximately evenly between these two mechanisms. Approximately half of the medals come from the same athletes ageing across successive Games, and approximately half come from later editions, drawing older first-time medallists (20). Neither mechanism can be isolated further in a series of this length. The age-period-cohort identification problem is structural rather than a matter of statistical power, and it does not yield a larger sample; it holds even when the whole population is observed (21–23). The rise is therefore reported as a description of what the podium has looked like across six editions, and not as evidence of the system that produced it.

A comparable pattern was documented outside fencing. In street skateboarding, the number of international competitors doubled among men and quadrupled among women within a decade of Olympic inclusion. The age at which the best performances occurred moved over the same interval, downwards for women, and towards a wider band for men (21). However, the direction of this study differed from that of the previous study. What recurs is how much an age pattern moves in the years just after a discipline enters the Olympic programme, and not the direction it takes.

Why the late twenties, in particular, rather than a younger or older band, is a separate question from the finding that both series converge there. One explanation is the competing demands of the sport. Speed has not declined far by that age, while tactical judgement has had time to build, so both demands are best met around then (7). This is a plausible explanation rather than a demonstrated one, however. These data show where medallists win, not the physical or tactical state that puts them there.

If a standardised preparation system had reshaped the age pattern, the change should have been evident across all sports, not in one weapon. Crossing the era with weapon discipline left the era effect intact, and the interaction was negligible. Foil, épée, and sabre differ in their scoring rules, physical demands, and tactical structures, and each has been subject to regulatory revision over the period, including the progressive electrification of scoring (1, 24). If the historical shift had been confined to one weapon, a change in the technical or regulatory character of that weapon would have offered a ready alternative explanation. Because all three weapons moved together, explanations based on a single weapon are difficult to sustain. This does not show that preparation was the cause because the demographic account considered predicts the same uniformity. The same period saw participation and success in Olympic sports broaden across nations (25); whether the national composition of fencing medallists shifted in step is consistent with this account, but was not tested here and remains for future work.

These findings address a gap in the literature on age and performance. Age of peak performance has been characterised across explosive-power and endurance events, but the available data were insufficient to describe mixed and skill events, which contain technical–tactical disciplines such as fencing (6). The present analysis describes one such discipline. This also suggests why the gap is difficult to close. Their framework ordered sports by event duration, which has little purchase on a sport whose demands are not primarily a function of how long the action lasts. The historical structure shown here adds a second reason, namely that the age pattern does not hold still, as the mean drifts downward while the spread contracts. A study reporting a single pooled mean and standard deviation would capture neither of these. It would report in their place a wide, stable-looking window that never existed in the sport's history.

The null result matters beyond the fencing. The 1972 boundary was defined in advance from historical accounts of the end of amateurism and not from age data (10, 11). If we had compared only the two periods, the 3.4-year gap might have been read as an effect of professionalization, which would have been incorrect. Sport science, state-supported training, and the commercial growth of the Games all ran through the same decades (10, 11, 19, 25), and no boundary separates them from professionalisation. This explanation therefore remains an inference (10, 11, 26).

A second account predicts the same contraction without reference to how the fencers were prepared. The number of fencers contesting Olympic medals grew across the period, while the number of medals available did not, and the range of nations from which medallists were drawn broadened (25, 27). The field grew larger and more uniform in standard, while the number of places remained fixed. Under these conditions, the observed range of any attribute, even weakly related to performance, narrows because the competitor at the edge of the distribution is displaced by one nearer its centre (28). A deepening field would also explain a wider pattern. The progression of the world's best performances came to a halt across many Olympic events towards the end of the 20th century, with most track-and-field and swimming events showing no further improvement after the early 1990s (29, 30). If that is correct, the fencer who won a medal at 45 or 50 in the early record was admitted not by a permissive preparation system but by a shallow field, and their disappearance requires no change in preparation at all.

The present design cannot determine which account is correct, and the two are not mutually exclusive: a preparation system that standardises entry into elite competition is one of the mechanisms by which a field deepens. Two features of the data bear on the question, without resolving it. First, contraction occurred in foil, épée, and sabre alike. This is consistent with a cause operating at the level of the sport rather than any one weapon. However, a growing field and a standardising preparation system both operate at that level; therefore, the result does not separate them. Second, the timing offers no leverage because the expansion of the field and the retreat from amateurism ran through the same decades. Distinguishing between the two accounts would require a measure of field size or competitive depth for each edition, which this dataset does not contain. What follows from the evidence is narrower than the preparation account alone: the contraction is real, and the preparation account, although consistent with it, is not the only account that is.

However, several constraints qualify these interpretations. First, the observations were not fully independent because some fencers won more than once. Most did not, so the analyses were predominantly based on distinct athletes. The dependence among the remainder is treated explicitly in the Results section, where the loss of effective information is quantified at approximately one-quarter. Non-independence, therefore, reduces the precision of these estimates without materially altering them.

Second, the two findings are based on limited data and should be interpreted accordingly. The female sabre trend was based on 18 medallists and was hypothesis-generating rather than confirmatory. The broader female era analysis was underpowered by design because women's Olympic participation is both shorter than men's and confounded with the staged introduction of each weapon. Its null result, therefore, cannot be taken as evidence that the male pattern is absent among women.

The population studied is the medal-winning one. The inclusion rule was held constant across the records so that the historical comparison would not absorb changes in how places below the podium were settled. This choice bounds the results. These data do not provide information on how the age distribution of fourth-place finishers, national squad members or club fencers has changed. The contraction reported here is a statement about the podium and not elite fencing at large.

Team events were excluded to avoid counting the same athlete twice for a single game, but this is not neutral for age. A fencer may be retained in a national squad for reasons that individual selection does not reward, including leadership and the value of accumulated competitive experience to less experienced teammates. Such a fencer can continue to win team medals even after the age at which winning an individual medal is realistic. The comparison between individual and team-only medallists was made for that reason, and its results do not support this concern. The excluded medallists were slightly older on average, and no more variable in age, and including them moved the dispersion ratio by approximately one part in a hundred. This analysis, therefore, describes the age at which individual Olympic fencing medals are won, and excluding team events, does not appear to have compressed the age distribution on which the dispersion finding rests (27).

A three-way model crossing era, sex, and weapon was not estimated. Women's events were introduced at different times (foil in 1924, épée in 1996, and sabre in 2004), which leaves many cells empty by construction, as no female sabre medallist could exist before the event was contested. A model spanning these cells would require values for combinations that never occurred, and its interaction terms would reflect the structure of the design rather than that of the sport. Thus, Era was analysed within each sex, and sex and weapon were analysed together separately; therefore, the female sabre trend is reported within its own discipline rather than as a term in a cross-weapon interaction.

Collectively, these results have implications at three levels. Theoretically, they identified the professionalisation of athlete preparation as a variable that age–performance research should treat as substantive rather than incidental. An age pattern may not be a fixed coefficient of a sport but a quantity that the surrounding development system can shift to. Characterisations of any discipline are, therefore, more defensible when read against its historical organisation. This places fencing, previously positioned as an intermediate case among combat sports based on aggregate age (5), within a dynamic rather than a static account.

Practically, the contemporary age pattern has settled in a band centred on 27 years, with half of all recent medals won between 24.1 and 30.8 years. A window of this width is relevant to periodisation and managing load across a career (31). Because the era effect held across foil, épée, and sabre, it can serve as a reference for pathway planning common to all three weapons rather than requiring a separate age template for each weapon. The upper tail also contracted, from a medal won at 50.0 in the earliest decades to 36.5 in the Modern Training era. Winning beyond the mid-thirties is now rare, so retention and resource cycles are better set against a late-twenties window than against open-ended career extension. For Olympic fencing medallists, this tempers the expectation that professionalisation simply lengthens competitive careers. These findings describe medal winners and should not be applied to talent identification or to sub-elite programmes.

Contextually, this case illustrates a methodological point. Examining a single discipline across the full span of its Olympic history reveals patterns that aggregated or single-edition analyses might miss. This principle extends beyond fencing: where a sport's age profile is suspected to have changed, considering the historical dimension is not merely an optional refinement but a requirement for accurately measuring the profile.

## 5 Conclusions

Across the modern Olympic era, age at medal winning in Olympic fencing behaved neither as a fixed feature of the sport nor as a quantity displaced by a single historical event. Among men, the mean age drifted downward slowly, by approximately one-third of a year per decade, and broke at no documented boundary, 1972 included; a model imposing such a break fitted the data worse than one that did not. The range of ages changed substantially. The spread of medal-winning age contracted by half again across the century, and the oldest medal-winning age fell from 50 to 36 years, gradually rather than abruptly, and in foil, épée, and sabre alike rather than in any one weapon. The age at which Olympic fencing medals are won has not changed. Winning one far from that age has become rare. Against that stable background, one series moved sharply in the opposite direction: female sabre, the most recently admitted discipline, whose medallists have grown older by approximately half a year for every year the event has existed. Based on 18 medallists, it is offered for monitoring rather than as an established result.

These findings contribute in three ways. Theoretically, they suggest that the organisation of athlete preparation is worth treating as a variable in age–performance research rather than as background, one that acts on the spread of the age distribution rather than only on its centre. Methodologically, they show that a historical category may name a real transition and still fail to describe the shape of the change across it; where an age pattern is thought to have moved, the form of that movement must be tested rather than assumed, because here a boundary comparison alone would have returned a confident and mistaken answer. Practically, the contemporary pattern is a band centred on 27.7 years, with half of all recent medals won between 24.1 and 30.8 years. This is common to all three weapons, which offers pathway planners a single late-twenties reference rather than a separate template per weapon, and indicates that winning beyond the mid-thirties is now historically rare. Whether these patterns hold below the medal-winning elite is a question for designs that reach below it.

## Data Availability

Publicly available datasets were analysed in this study. The data can be found here: https://www.olympedia.org/sports/FEN.
