# Supplementary material for: Olympic fencing medallists are increasingly clustering around an age that has hardly changed (1896–2024)
Source: Front Sports Act Living. 2026 Oct 7;8:1938745. doi: 10.3389/fspor.2026.1938745 (PMC13683601; doi:10.3389/fspor.2026.1938745)
Supplement: Supplementary file 1 [file Table1.docx]

Supplementary Material

# 1 Supplementary Data

The compiled dataset, the audit log documenting every correction applied to it, and the analysis code are available from the corresponding author on reasonable request. The two tables below report analyses referred to in the main text and are supplied here rather than in the article to keep the number of display items within the journal limit.

# 2 Supplementary Tables

**Supplementary Table 1**

*Age at Medal Winning by Era: Individual Medallists and Team-Only Medallists*

| Era | Team-only medallists | | | Individual medallists | | |
| --- | --- | --- | --- | --- | --- | --- |
|  | *n* | *M* | *SD* | *n* | *M* | *SD* |
| 1896–1912 | 56 | 31.80 | 5.85 | 37 | 29.92 | 8.23 |
| 1920–1936 | 178 | 33.40 | 6.72 | 56 | 30.27 | 5.87 |
| 1948–1968 | 231 | 30.04 | 5.93 | 72 | 30.91 | 6.33 |
| 1972–2000 | 324 | 26.78 | 4.29 | 102 | 27.16 | 4.16 |
| 2004–2024 | 245 | 28.42 | 4.61 | 108 | 27.73 | 4.90 |

*Note.* Both sexes pooled. Ages are decimal. Team-only totals are 1,034 medal-winning appearances by 766 athletes; individual totals are 375 observations from 282 athletes.

**Supplementary Table 2**

*Breakpoint Sensitivity for the Male Age Trend, 1896 to 2024*

**Panel A.** Every Olympic year as a candidate single knot, ranked by AIC.

| Rank | Knot | ΔAIC | *R²* |
| --- | --- | --- | --- |
| 1 | 1904 | 0.00 | .127 |
| 2 | 1908 | 7.83 | .100 |
| 3 | 1912 | 11.08 | .089 |
| 4 | 1924 | 11.58 | .087 |
| 5 | 1920 | 11.62 | .087 |
| 6 | 1928 | 12.87 | .082 |
| 7 | 1932 | 14.24 | .078 |
| 8 | 1948 | 14.36 | .077 |
| 9 | 1936 | 14.54 | .077 |
| 10 | 1952 | 15.07 | .075 |
| 11 | 1956 | 16.69 | .069 |
| 12 | 1960 | 18.13 | .064 |
| 13 | 2012 | 18.75 | .062 |
| 14 | 2008 | 18.78 | .061 |
| 15 | 2004 | 18.89 | .061 |
| 16 | 1964 | 19.20 | .060 |
| 17 | 2016 | 19.20 | .060 |
| 18 | 2000 | 19.28 | .060 |
| 19 | 1996 | 19.46 | .059 |
| 20 | 1968 | 19.78 | .058 |
| 21 | 1992 | 19.96 | .057 |
| 22 | 1972 | 20.24 | .056 |
| 23 | 1988 | 20.27 | .056 |
| 24 | 1984 | 20.48 | .055 |
| 25 | 1976 | 20.55 | .055 |
| 26 | 1980 | 20.61 | .055 |

*Note.* Male medallists, *N* = 261. A straight line with no knot has ΔAIC = 18.62 (*R²* = .055). Of the 26 candidates, 14 fit worse than this, and every candidate that improves on it lies at 1960 or earlier. The amateurism boundary at 1972 ranks 22nd.

**Supplementary Table 2 (continued)**

**Panel B.** Models built on the four documented historical boundaries.

| Model | *k* | ΔAIC | *R²* |
| --- | --- | --- | --- |
| Knot 1914 | 3 | 0.00 | .089 |
| Knots 1914 + 1939 + 1972 | 5 | 0.62 | .101 |
| Knot 1939 | 3 | 3.37 | .077 |
| Linear, no knot | 2 | 7.63 | .055 |
| Knot 2000 | 3 | 8.30 | .060 |
| Knot 1972 | 3 | 9.25 | .056 |
| Intercept only | 1 | 20.34 | .000 |

*Note.* Male medallists, *N* = 261; *k* is the number of estimated parameters. ΔAIC is computed within each panel separately. Adding all three of 1914, 1939, and 1972 improves on none of them individually.
